# “I have always found the whole area a minefield”: Wikidata, historical lives, and knowledge infrastructure

**DOI:** 10.1007/s42803-024-00090-5

**Published:** 2024-12-23

**Authors:** James Baker, Ammandeep K. Mahal

**Affiliations:** University of Southampton, Southampton, United Kingdom

**Keywords:** Wikidata, Infrastructure, Knowledge, Classification, Historicity

## Abstract

The rise of Wikidata represents a quiet revolution in knowledge infrastructure. This paper enquires into this knowledge base as an infrastructure and considers the implications of its centrality within our contemporary knowledge ecosystem. Rather than read Wikidata at scale, we employ of a narrow frame through which to explore the ideologies Wikidata has adopted and reproduces. This frame is Beyond Notability, a knowledge base that seeks to document women’s work in archaeology, history, and heritage between 1870 and 1950 through original archival research. Beyond Notability draws on and responds to the Wikidata data model, and this paper emerges from our experiences interacting with Wikidata to produce linked data biography. In foregrounding the tensions between historically specific phenomena and classificatory logics, our work stresses the value of using practice-based ontology development to investigate large-scale knowledge infrastructures at a time when the fabric of knowledge is at stake.

*It might have been otherwise* (Star, 1990)

The riseof Wikidata represents a quiet revolution in knowledge infrastructure. Launched in late-2012, Wikdata is a knowledge base, a source of linked open data on people, places, things, and concepts, designed to be read and edited at scale by both people and machines. Wikidata is a central node within contemporary knowledge infrastructure. It is a repository and federator of subject and domain specific identifiers, from ISNI for people or IMDb for films, to Basisklassifikation for library classifications or swMATH for mathematical software. It is a source of knowledge that is amplified by web content, digital assistants, and services such as Google’s Knowledge Panel. It is a platform for knowledge production and for the machine-readable communication of research results (Rutz et al., 2022). It is a source of data enrichment, verification, and linkage (Candela et al., 2023). And it is a model for the creation of new knowledge graphs, with Wikibase – the software developed for and used by Wikidata – underpinning platforms for exploring the lives of enslaved individuals, geographical metadata, and the work of many libraries and research institutes.

This paper enquires into Wikidata as an infrastructure, what that infrastructure produces, and the implications of its centrality within knowledge infrastructures, systems, and wider society. We are guided in this work by scholars of infrastructure and critical data studies, work that collectively imagine these datafied knowledge infrastructures as embodiments of (not necessarily harmonious) community conventions and standards, and the classificatory logics they deploy as the products of power and labour that is always relational and consequential (Agostinho, 2019; Borgman, 2015; Bowker & Star, 2000; Thylstrup et al., 2021).

As one of the Wikimedia Foundation’s family of sites – the most well-known of which being Wikipedia – Wikidata is maintained by volunteer community labour and open collaboration using a wiki-based editing system. As with any community, this community is particular and far from uniform.^2^ Previous studies seeking to understand who produces knowledge on Wikidata have focused on community structure, the relative heterogeneity of ‘leaders’, ‘contributors’, and bots, and the relationship between experienced editors and the ontological quality of the edits they make (Piscopo, 2019; Piscopo & Simperl, 2018). These studies are framed by research into the Wikipedia editing community, in particular the well documented underrepresentation of historically minoritised communities and demographics within that community (Graham et al., 2015; Sengupta & Bouterse, 2017; Tripodi, 2021). Recent work suggests that just 10% of Wikipedia volunteers identify as a woman or non-binary and that only 20% of information found in the encyclopaedia is created on or by people from the Global South (Causevic et al., 2020; Sengupta, 2021).

Exploring the conventions, standards, and related work that helps to shape Wikidata as a large-scale classificatory infrastructure is then a vital task. The infrastructures of the internet do not look or sound like most people in the world (Sengupta, 2021). And there is a wealth of evidence available relating to how classification, datafication, and machine processing can further minoritise already minoritised communities, especially so when that the work is undertaken without sufficient representation of and from those communities (Bender et al., 2021; D’Ignazio & Klein, 2020; Lavorgna & Ugwudike, 2021; Noble, 2018). Though such work is vital, it is also a formidable task. Wikidata contains almost 110 million items (an item being the ‘subjects’ and ‘objects’ that make up subject, predicate, object triples), over 11,000 properties (the ‘predicate’ that binds those triples), more than 1.7 billion page edits, and close to 12 billion words of community commentary and discussion (Pellissier Tanon and Suchanek, 2019).

Rather than attempt to read Wikidata at scale, an approach common in the human-computer interaction and network studies literature, we employ of a narrower frame through which to enquire into Wikidata-as-infrastructure, to tease out the ideologies it has adopted, continues to take for granted, and is likely to reproduce. As Tom Wallaert and Guido Roumans have demonstrated, narrow frames of study can provide deeper understandings of complex knowledge systems (Willaert & Roumans, 2020). Beyond enabling the identification of errors and discrepancies between such knowledge systems, these frames open up questions of consensus building and classificatory structures. Our narrow frame is another knowledge base, specifically *Beyond Notability*, a knowledge base that uses original archival research to document women’s work in archaeology, history, and heritage between 1870 and 1950 (Baker et al., 2024). The *Beyond Notability* ontology both draws on and responds to the Wikidata model: at times aligning directly so as to enable ease of cross-querying (e.g. reusing Wikidata properties indicating family relations such as ‘mother’, ‘father’, or ‘spouse’); on other occasions deviating so as to reframe Wikidata properties towards the active, historicised, and biographical focuses of *Beyond Notability* (e.g. the Wikidata property ‘residence’ (P551) becomes the property ‘resided at’); as well as creating new properties specific to the field of study and archival records under examination (e.g. developing ‘election to SAL proposed by’ as a means of capturing the networks that supported women seeking to enter spaces such as the Society of Antiquaries of London). This article then emerges from our experience interacting with Wikidata, the ways we have questioned Wikidata’s assumptions and conventions, and in particular how concepts, people, and worldviews are skewed to fit its worldview.

We proceed in three parts. First, as a precursor to two specific case studies, we consider the presentness and atemporal characteristics of many Wikidata statements used to construct linked data biography. Here we focus on Wikidata properties and reflect on what their characteristics impose on relationships between people, spaces, and objects. We argue that by favouring event-based temporality, what Fernand Braudel famously saw as proverbial surface ripples (Braudel, 1972), the Wikidata model limits space and scope for representing the rich, subtle, and granular conceptual change we encounter in our archival research. Second, the article examines Wikidata’s approach to ascriptions of ethnicity, race, and citizenship. We explore how the approach used contributes to the omission of identity expression and colonial geopolitics from Wikidata biographies and to a normalisation of unmarked ‘whiteness’. Third and finally, we investigate Wikidata’s collapsing of sex and gender, normalisation of gender ascription, and inattention to how gender varies geographically and temporally. Taken together we contribute to understandings not only of Wikidata and its significance in present day knowledge ecologies but also of how meaning is made on community platforms like Wikidata. Importantly, we also demonstrate how historical specificity can be brought to bear on modern information infrastructures in ways that can support both more accurate representation of the knowable past and reshape the possible futures we create.

Throughout we are guided by histories of ‘professional’ women in late-nineteenth and early-twentieth century Britain. Together these histories have explored how women’s work in archaeology, history, and heritage was constructed and imagined in these male coded arenas and the ways that women worked against this coding to craft particular forms of intellectual visibility (Hill, 2016; Stevenson, 2019; Thornton, 2018), as well as articulating the wider societal constraints that shaped professional women’s lives, career development, and authority in the workplace (Glew, 2016; Langhamer, 2017; McCarthy, 2021). Helen McCarthy in particular, has illuminated how in the second half of our period, a significant attitudinal shift took place that reconfigured the landscape of paid work for women and its relationship to traditional roles of motherhood and wifely household management. It is shifts of this nature that, we argue, are not adequately captured by Wikidata. In foregrounding the tensions between historically specific phenomena and classificatory logics, our work stresses the value of using practice-based ontology development to reflexively investigate and critique large-scale knowledge infrastructures in the age of platform web technologies, and at a time when the very fabric of knowledge is at stake (Gebru et al., 2023).^3^

## Present and ahistorical

The *Beyond Notability Knowledge Base* (hereafter, *Beyond Notability*) documents women’s work in archaeology, history, and heritage between 1870 and 1950. Evidence in records at the Society of Antiquaries of London, the Royal Archaeological Institute, and related archives is transformed into subject-predicate-object triples that record information such as the dates on which people were elected as fellows of scholarly societies, the committees or councils they joined, where they were educated and what they studied, the people and bodies they corresponded and collaborated with, the excavations they coordinated, and/or the roles – paid or otherwise – they held at institutions. The data model developed to marshal this data uses a capacious and expanded conception of work that recognises the shifting status of and perspectives on certain forms of labour in our period and their relationship to intersections of class, gender, race, religion, and locale. It is also a data model that locates work in relation to people and biography – to the cultural and economic capital provided by family and marriage, to social and emotional infrastructures of collaboration and friendship, to life events that take a person away from work or provide a new lens on its value.

In operationalising this conception of work as linked data, *Beyond Notability* diverges from Wikidata-as-infrastructure in crucial ways. For example, on Wikidata the property ‘employer’ (P108) is used on over 1.9 m item pages (Wikidata, 2023), approximately 20% of all biographical entries for people.^4^ These usages represent the relationships between people and the organisations they worked for, typically with qualifiers that provide temporal precision and describe the roles individuals performed: for example, that Neil Armstrong worked for NASA between 1962 and 1971 as an astronaut; or that between 1978 and 1990 Angela Merkel worked for the Zentralinstitut für Physikalische Chemie as a research fellow (*Wikidata*, n.d.; *Wikidata*, n.d.). An alternative set of relations are offered by the Wikidata property ‘position held’ (P39), foregrounding the relationships between people and what they did rather than who they did it for. However, most of the roughly 820,000 uses of P39 express the relationships between people and civic office: bishoprics, mayoralties, of justice, or in legislative assemblies. Wikidata-as-infrastructure then reproduces a particular kind of labour relations, one that narrowly defines work through employee-employer dynamics and normalises Fordist models of socio-economic conditions and wage labour.

This is not a data model that can adequately represent the forms of labour women experienced in archaeology, history, and heritage during the late-nineteenth and early-twentieth centuries. In response, *Beyond Notability* models work in two ways. First, where evidence of formal and/or longstanding labour relations either existed or can be reasonably assumed to have existed, the property ‘employed as’ (P105) is used to represent the work women did as *inter alia* secretaries, librarians, editors, lecturers, directors, principals, curators, and cataloguers. Second, in cases where labour was more likely to have been undertaken on occasional, freelance, informal, voluntary, or honorary bases the property ‘held position’ (P17) is used to represent not only the often insecure or unwaged work that women did as secretaries, librarians, editors, lecturers, directors, principals, curators, and cataloguers, but also as treasurers, record agents, chairs, local secretaries, and research assistants, a proliferation of work-like roles that are not captured adequately by presentist models of labour relations. The organisation for whom the work was conducted is then expressed in each statement as a qualifier, using ‘employer’ (P18) for ‘employed as’ statements and ‘of’ (P78) for ‘held position’ statements, to distinguish between – for example – the position that Edith Bradley held in 1892 as Secretary of the Association of Women Pioneer Lecturers and Dorothy Nancy Stroud’s employment as Assistant Curator at Sir John Soane’s Museum in the early-1950s (*Beyond Notability*, n.d.; *Beyond Notability*, n.d.).

The implications of this shift in emphasis becomes clear if we focus on the modelling of an individual working life. Joan du Plat Taylor was a mid-twentieth century maritime archaeologist who made substantial and pioneering contributions to the study and excavation of underwater sites. The Wikidata entry for Taylor is typical in its use of community conventions for recording work: her occupation is stated to have been an ‘archaeologist’ and her employers the UCL Institute of Archaeology between 1945 and 1970 and the Nautical Archaeology Society between 1972 and 1980. On *Beyond Notability* the entry for Taylor provides different points of emphasis: that she held the position of Assistant Curator at the Cyprus Museum, that she was employed as a Librarian by the University of London’s Institute of Archaeology (the Institute joined UCL in 1986), that in 1948 she spoke at the Society of Antiquaries of London on the subject of her work in Cyprus with Veronica Seton-Williams. The point here is not to focus on the granularity or quality of data compiled by the two knowledge bases: a domain specific knowledge base like *Beyond Notability* is intended to capture a level of complexity not possible – or desirable – in a general-purpose knowledge base like Wikidata. Rather the point is to focus on how the organisation of the data differ between the two infrastructures. And what we find when comparing entries for women like Joan du Plat Taylor is that the structuring of Wikidata around notable and temporally intelligible instances of wage labour impose onto the record of their working lives a structure that decentres agency and expertise and that flattens historical specificity.

Such temporal flattening extends to how Wikidata represents the life course. In late-nineteenth to mid-twentieth century Britain the workplaces women encountered were male coded and – whilst subject to change – often structured women’s roles under the assumption that women would hold them temporarily, that they would – by convention or requirement – change their role or cease working when newly married or when rearing children. For example, Tessa Verney studied History at UCL between 1911 and 1914, married the archaeologist Mortimer Wheeler in 1914, gave birth to their son Michael in 1915, moved to Cardiff after the Great War when her husband obtained a post at the National Museum of Wales, supervised major excavations in the 1920s (at Segontium, Gaer, and Caerleon), returned to London in 1926, was elected a Fellow of the Society of Antiquaries of London in 1928 (at which point the field specifying ‘occupation’ on her election candidate certificate was left blank), and held the position of Secretary of the Institute of Archaeology in 1936. Her work in archaeology was then entangled with life events: education, marriage, parenthood, war, relocation. The Wikidata data model and community conventions are adequate – if imperfect – for representing these events with one important exception: for childbirth. That is, unless a person had a child who was themselves sufficiently notable to warrant a Wikidata entry, and in turn a date of birth, all other acts of childbirth on Wikidata are represented by ‘number of children’ (P1971), a property that captures the number of children a person had – or has had so far – in their lifetime. This view of a life from an atemporal perspective disables our ability to see across and through life events, untethering – for example – the temporal connections between Olwen Brogan (1900–1989) raising four children and her education and employment at UCL, her marriage, her service on the Council of the Royal Archaeological Institute, her election as Fellow of the Society of Antiquaries, and her receipt of an OBE (*Wikidata*, n.d.).

In short, Wikidata’s use of statements about children is inattentive to how parenthood relates to other events in the lifecourse, to the intersections between age, parenthood, gender roles, marriage, and social class. This in part can be explained by what is achievable through community and voluntary production of data: a statement listing the number of children someone had will take considerably less time – and research – to implement than multiple statements recording the dates on which multiple children were born. But by taking an atemporal viewpoint in some cases, and by aligning with a set of present-oriented worldviews in others, the model opens limited space for the rich, subtle, and granular conceptual changes required when researching and representing complex historical phenomena. As we turn now to examine how the Wikidata community models ethnicity, race, and citizenship, the impact of these limitations come into sharper focus.

## Race, ethnicity, citizenship

*Beyond Notability* is creating linked data biographies. In so doing we model and test our approach against other biographical genres and sources of personal data – the identity card, the obituary, the autobiography, the letter of recommendation. Semantic triples can in theory capture the rich tapestry of assigned and reported selfhood present in such sources. In reality linked data biographies tend to be constrained to the kinds of data we might expect to provide in a passport application, to a new employer, or on a landing card. However, whilst contemporary citizens are routinely expected to describe what we conceive as our ethnicity, ethnic identity, and/or racial background (or, in some cases, state our preference to not provide such information, to resist the exercise altogether), these data are routinely absent from knowledge bases like Wikidata. And where they are present, the Wikibase model variously blurs and conflates their historical, experiential, and expressive complexity.

Consider, for example, a biographical representation of Stuart Hall, the cultural theorist whose career was preoccupied with the use of identities, unsettled representations of race and ethnicity (neither of which categories Hall was fond it), and how those processes produce one another. The *Oxford Dictionary of National Biography* entry for Hall, written by Martin Jacques, begins:

**Hall**,** Stuart McPhail** (1932–2014), cultural theorist and political commentator, was born on 3 February 1932 in Kingston, Jamaica, the son of Herman McPhail Hall, accountant, and his wife, Jessie. He was of mixed African, Scottish, and Portuguese descent. He had a brother and sister, both of whom were older than him [.] Hall grew up in Kingston. His brown-skinned father rose to become the chief accountant of the Jamaican subsidiary of the American giant United Fruit. His fair-skinned wife (Hall’s mother) never worked outside the home but treated the family as her personal fiefdom. Hall, for his part, had by far the darkest skin in the family. (Jacques, 2018).

Here precise details of place, descent, and physical appearance are central to a narrative biographical portrait, and combined with a photograph of Hall those narrative details intersect to produce a nuanced and sensitive sense of his personhood in ways that text and image could not in isolation. The Wikidata item for Hall records similar biographical information to Jacques, with semantic triples replacing narrative biography to elucidate Hall’s education, professional activities, employers, awards, marital relations, and death (*Wikidata*, n.d.). The entry also, like Jacques’ biographical introduction, records where and when Hall was born. What it omits are Hall’s parental ties to that place of birth, the ‘mixed African, Scottish, and Portuguese descent’ Jacques foregrounds, the colonial entanglements his descent invokes, and any – non-pictorial – information about his physical appearance or those of his family.

Ontologies of physical appearance are closely associated with racial (and racist) science, with certain groups of Europeans as the proper producers of (scientific) knowledge, with political languages and cultures mired in race-thinking (Bowker & Star, 2000; Gilroy, 2000; Seth, 2009). The Wikimedia family of sites explicitly seek to prevent such practices (Wikipedia, 2004). On Wikidata this is achieved using conflict constraint warnings. And so whilst ‘light skin’, ‘dark skin’, ‘olive skin’, and other skin type items all exist in Wikidata as instances of ‘human skin color’ (Q853516), the ontology created and maintained by the Wikidata community uses conflict constraints to flag – and in turn, remove – instances where editors have made statements of ‘colour’ (P462) in relation to the physical appearance of real people.

Similarly, the Wikidata property for ‘ethnic group’ (P172) describes the permissible uses of the property in heavily caveated terms:

subject’s ethnicity (consensus is that a VERY high standard of proof is needed for this field to be used. In general this means 1) the subject claims it themselves, or 2) it is widely agreed on by scholars, or 3) is fictional and portrayed as such).

As a result of this ethos fewer than 110,000 Wikidata items contain statements that use ‘ethnic group’ as a property (Uses of P172, n.d.), and at any given time – between the regular bot driven deletion sweeps – roughly half of these violate the property referencing constraint (*Wikidata*, n.d.). As with physical appearance, ethnicity then sits firmly outside of the core Wikidata biographical ontology.

The point we make here is neither that skin colour and ethnicity should be active categories within Wikidata, nor that the juxtaposition of Wikidata with Jacques biography of Hall is intended to imply that the former should give way to the latter. Indeed, our ontological work on *Beyond Notability* is shaped by not giving credence to the notion that ethnicity is a knowable fact, and we seek to avoid normalisation of unmarked whiteness by – say – creating categories for minoritised cultural groups (Mirzoeff, 2023). We determine that as relational constructs, such categories are too culturally unstable and spatio-temporally variable to be of practical use in statement-led formulations. And whilst not including such data risks further occluding the presence of Black British people from British history and contemporary life (Bressey, 2006), as well as creating the conditions for users to assume whiteness in the data, we determine that were they to be used, such use would impose essentialist and positional logics, and embed into knowledge bases like Wikidata locally inscribed regimes of truth in ways that Hall refuted, a deeper presentism to an already presentist infrastructure.

Rather, we assert that if narrative biography cannot be easily rendered as linked data, (Willaert & Roumans, 2020), then linked data should not be asked to stand for narrative biography and – in turn – lived experience when those linked data are used as data and in aggregate. And yet this is precisely how Wikidata has come to represent ethnicity via citizenship. The *ODNB* biography of Hall is again of use to us here. Jacques writes:

Hall never regarded himself as English. Indeed he was to think himself less and less English as the years went by. But nor did he think of himself as simply Jamaican, for he had left the island when he was nineteen, never to live there again. He came to think of himself as being of both and neither, of being ‘here’ and ‘there’, of being diasporic. He rejected the idea of identity as fixed, arguing that it was always in a process of constant change. In his own words, ‘identity is not settled in the past but always also oriented towards the future’ (Jacques, 2018).

On Wikidata, such complex relationships between geopolitics, colonial history, and migration are represented by statements that make use of the property ‘country of citizenship’ (P27); in the case of Hall, he is represented as having been a citizen of both the ‘United Kingdom’ and of ‘Jamaica’. Neither of these statements is untrue, and the repeatability of predicates within linked data ontologies does allows for Hall’s dual and unsettled relationship with belonging to be invoked, if in ways that are inattentive to temporal change or colonial and colonised experience. And yet the clarity of both statements is weakened by slippages between country, state, and nation in surrounding statements. For example, on Wikidata, both ‘United Kingdom’ (Q145) and ‘Jamaica’ (Q766) are given as instances of ‘country’, ‘sovereign state’, and ‘island nation’. This plurality muddles the proverbial waters, producing when used in P27 statements something Hall keenly opposed: a social usage of citizenship as a rough proxy for fixed ethnic nationality, a usage that is misaligned with diasporic experience, with shifting expressions of, relationships to, and constructions of nationhood, belonging, custom, place, and history; structures of feeling which Hall experienced and felt (Hall, 2017: 175).

These P27 statements are therefore not unproblematic assertions of legal citizenship, rather they function as ascriptions of identity. The talk page for ‘country of citizenship’ documents the ways that the Wikidata community have struggled to reconcile this implicit contradiction, and to maintain norms through which the property can be used without onerous standards of proof. Indeed, the property has been fraught since inception. Initially conceived as ‘Nationality’, its first English language revision changed the property label to ‘Country of Citizenship’ (Wikidata, 2013a). Justifying the decision, one editor wrote in February 2013:

In the spirit of having the most unambiguous property label, I’m changing “Nationality” to “country of citizenship”. “Nationality” has several possible meaings [sic], and, perhaps more importantly, several possible phrasings - if I were to have an item, would it say my Nationality were “America”, “American”, or “Americans”? Imagine how much more complicated it would be if I lived elsewhere, or had changed citizenship… this way, if there’s a need, we can also have a “country of residence”, “country of birth” (though we already have a “place of birth”), “previous countries of residence” - whatever. But “Nationality” is far too vague (PinkAmpersand, 2013: 27).

Unambiguous it may have seemed, but the presentist mindset of the property rapidly became apparent. In March 2013 the editor Zolo noted that the property description “the object is a sovereign state that recognises the subject as its citizen” was unsuited to pre-modern (European) models of citizenship, citing the example of Dante Alighieri, in whose time Italy was not a sovereign state. And yet Zolo also felt the property should still be used for historic figures, because ‘it sounds sort of obvious to most people that Dante is Italian’ (Zolo, 2013).

In the case of Dante, a solution was found with the introduction of the ‘historical country’ (Q3024240) class, and its use alongside the class ‘sovereign state’ (Q3624078), to describe historic entities such as the ‘Republic of Florence’. But a sense of the property’s temporal ambiguity remained unresolved, resulting in the community raising further qualms: how to deal with citizenships that were created or reorganised during a person’s lifetime (such as Hall’s); what to do with social organisations that existed outside of imperial notions of citizenry; how to describe people whose nationality was declared for them through conflict or annexation; how to manage items for countries that collapse various configurations of a state into its labels and aliases; and – crucially – problems associated with slippages caused by (narrative) statements of nationality on Wikipedia being used for (triple) statements of citizenship on Wikidata (Wikidata, 2015; Zolo, 2013). In short, the complexities that a plurality of scholars, from those working on socio-technical media aesthetics to historians of early modern identity, have described as having been central to regimes of identity formation (Dhaliwal, 2022; Groebner, 2007) – that there is always a gap between a person and their papers, that people do not control the disciplinary systems that construct their identity, and that identification is a process shaped by history, circumstance, and purpose – have thwarted the Wikidata community’s pursuit of clarity. As the editor Jheald put it in July 2018:

country of citizenship (P27) has been kicked backwards and forwards a lot, as to whether it should be read narrowly as connecting somebody to a particular state that existed in their lifetime, or should be read more widely to allow a painter to be designated eg French or Italian or Flemish at a time when those states may not have existed in their current form, or even (Flemish) not necessarily existed as independent states granting ‘citizenship’ at all. I have always found the whole area a minefield, that I wish somebody would definitively clean up (Jheald, 2018).

This central tension remains, and a lazy consensus has won out: statements of citizenship *should* record a person’s citizenship as associated with states as they existed in their lifetime, but *in reality* many such statements record something closer to an assertions of citizenship through proxies of nationality. In turn, Stuart Hall, who was born in Jamaica at a time when the island was a British Colony, is described on Wikidata as a citizen of ‘Jamaica’ rather than of the ‘Colony of Jamaica’. Which of these is correct is not the point: rather we observe an aspect of Hall’s identity expression being assigned under the auspices of a property supposedly used to assert legal citizenship; we observe identity making processes being remade and reamplified through Wikidata, its infrastructure, and its communities.

A final reflection. The Wikidata entry for Hall states that his ‘sex or gender’ (P21) is ‘male’ (Q6581097). The statement is evidenced by assignments of ‘sex or gender’ on other knowledge bases. It is unclear whether Hall identified as male, a man, a guy, masc or any of the other words Wikidata collapses the term ‘male’. But it is clear that various elisions of legal sex, gender markers, and identity expression have taken place in order to ascribe this marker of gender. Significantly, this consolidation of the gender binary takes places on Wikidata without property constraints and is not subject to the same standards of proof required to assert a person’s ethnic identity. Having outlined the constraints created by the Wikidata community to prevent ascriptions of ethnicity, the confidence with which editors ascribe gender to individuals – to which we now turn – is jarring.

## Sex and gender

The property ‘sex or gender’ (P21) is defined in Wikidata – at the time of writing – as follows:

sex or gender identity of human or animal. For human: male, female, non-binary, intersex, transgender female, transgender male, agender. For animal: male organism, female organism. Groups of same gender use subclass of (P279).

This property is used in over 8.32 million statements, 6.25 million of which take the object ‘male’, 2 million of which take the object ‘female’. Fewer than 5,000 statements use P21 to describe trans, non-binary, intersex, third gender, or other related gender identities (Wikidata Query Service, n.d.), many fewer uses than for non-human animals. The significant under-representation of both women and trans, non-binary, intersex, and third gender people in Wikidata is of course notable. But *who* is represented is perhaps not the central issue. Rather it is *how* they are represented, how the resilience of the gender binary and a collapsing of sex and gender combine to create a contestable classificatory logic that is unsettled by encounters with mismatches, with those at the margins of those logics – and as is so often the case with such systems, we see Wikidata’s classification system for sex/gender torque and twist to accommodate those at the margins of its logics, undermining the legitimacy of the system and underscoring its inadequacies (Bowker & Star, 2000: 223).^5^

P21 was contested from outset. On 4 February 2013, when the first set of statements were launched on Wikidata, ‘gender’ was created as Wikidata’s twenty-first property, restricted to taking one of two objects: ‘maschio’ or ‘femmina’ (Wikidata, 2013b). The next day it gained a third constraint via the English language description ‘MUST BE ONE OF: male (Q44148), female (Q43445), or intersex (Q1097630)’ (Wikidata, 2013c). By the end of the day, the property had been renamed ‘sex’ (sex (P21), 2013). Over the course of 2013, the community development of the property was guided by this logic of biological sex: the description acquired constraints for the sexes of non-human animals; English, French, and German descriptions of the property were aligned; and a range of aliases were attached to the property, including ‘gender’, ‘gender identity’, ‘gender expression’, and ‘biological sex’ (Wikidata, 2013f). Then on 26 December 2013, Filceolaire – a founding Wikidata editor – made a decisive edit: changing ‘sex’ to ‘sex (or gender)’ (Wikidata, 2013e). A month later the brackets were removed, resulting in the formulation – ‘sex or gender’ – that remained in place throughout the period in which Wikidata grew into a central node in our global knowledge infrastructure (Wikidata, 2014).

The move from ‘sex’ to ‘sex or gender’ was not the result of either rogue, quiet, or illicit editing. Rather the evolution of P21 between early-2013 and early-2014 was accompanied by substantial debate within the Wikidata community. The tone of that debate was broadly polite and constructive, with an instinctive and opinionated tenor characteristic of virtual communities – the talk pages show a community discussing concepts, examining opinions, cross-talking, apologising, chatting; a polyphony at odds with the clean, always-already objectivity of a Wikidata property description. Two exchanges from this period are notable. The first cascaded from a question by an editor about mismatches between classifications: ‘What do you enter when sex and gender do not agree?’, Kaldari asked. Their fellow editors responded with comments that variously indicated confusion (‘When do we have that problem? I am maybe lost in translation now’), biological essentialism (‘I do think we need to establish a time frame for transexuals’), and a desire for the community to read around the subject, to educate (Wikidata, 2013h). Here then we find the imperatives to classify and to ascribe aspects of identity expression encounter cases that trouble those imperatives, if not sufficiently for those imperatives to be challenged. The second notable exchange – a few days later and again prompted by Kaldari – related to a proposal to revert the label for P21 back to ‘gender’. Here the community discussion centred on thresholds for establishing knowledge, reasonable inferences, and proxies for classification, as well as whether or not to have separate properties for ‘sex’ and ‘gender’ (Wikidata, 2013g: 1). For Kaldari, ascertaining a person’s gender, or at least socio-cultural ascriptions of their gender, was just as – if not more – straightforward as ascertaining their ‘sex’, and hence should be the criteria by which P21 statements were made. As they wrote:

By ‘gender’, I mean the gender someone is publicly known as. I don’t agree that it’s easier to know someone’s sex than their gender. If someone is a notable transgender person, it’s generally known which gender they identify with. It is not always known, however, if they have actually had sex-reassignment surgery. And it is very rarely known if someone is actually intersex since intersex people normally pass as male or female. Either way, it is rather awkward and clinical to classify people by ‘sex’ instead of ‘gender’ (Kaldari, 2013).

The proposal was – however – rejected. This was in part because some members of the community did believe that ‘sex’ was easier to define – as one editor wrote ‘Yes, we know the sex of historical figure [sic]. Basically, you were not allowed to be King of France, or to vote in the 19th century or to marry a female if you were not a biological male. Your gender has nothing to do with it’ (Wikidata, 2013d). But the proposal to change P21 from ‘sex’ to ‘gender’ was also rejected because an uncomfortable consensus was emerging, one that viewed gender ascription, gender presentation, and editorial presumptions about biology as all sufficient to state that someone felt ‘male’ of ‘female’.

Within months members of the community began to treat the issue as resolved. ‘People need to understand’, remarked the editor Tobias1984 in November 2013, ‘that our male and female category is a mix of biology and culture’ (Tobias1984, 2013). In July 2014 Filceolaire – reflecting on discussions the previous year – noted that ‘we can tell what gender they publicly express and from that we can guess at their biology’ (Filceolaire, 2014). And the same month, the editor Emw claimed to be expressing a community at ease with an apparent consensus when they wrote:

We see that there is little appetite in the Wikidata community, at least with this property, for a clear separation of concerns. Not only does P21 conflate sex and gender, it also conflates sex and biological classification (Emw, 2014).

This uneasy resolution would endure. And as Wikidata matured and grew this polyphonous consensus was fused with an apparent desire for simplicity. As one editor noted in 2019 at the start of a discussion around design criteria for the WikiProject LGBT community’s work on Wikidata:

There are a lot of different concerns when it comes to gender and sex. On the one hand there’s a need to have an idealistic way to model gender. On the other hand there are other needs as well. The statements we have should for example make it easy for automated tools to know what pronouns they should use when describing a given person (Wikidata, 2019).

The presence of the gender binary at the heart of Wikidata’s biographical infrastructure was then the result of prolonged engagement with a minority of biographical subjects whose lives were at odds with Wikidata’s classificatory logics, and considerable deliberation over what about a person is knowable. And once that binary was settled on, it was entrenched by community members who retained and reaffirmed their knowledge of those debates, who thought that whilst P21 was not ideal it was of greater utility to the ontology in a simpler – fudged – ‘sex or gender’ form.

The problem is how that knowledge seemingly ebbed away from P21 over time, how the unsettling, the mismatch with reality resolved into a community consensus whose polyphony is opaque, and that looks – from the outside, used at glance, as most classificatory logics are – like just another assertion of the gender binary. This has had consequences, most notably in that assignments of gender and/or sex *to* subjects is the dominant mode in which Wikidata is imagined and used. For example, Wikidata statements about subjects are often made by bots: human directed assignments of automated knowledge making that are designed and deployed in order to achieve consistency and order across Wikidata. In April 2021, the editor Crystal Clements observed bots working in ways that they argued were ‘heavily biased towards cisgendered people, especially those in cultures where unisex names are commonplace’ (Clements, 2021). In February that year Clements had, in the process of adding new biographical entries to Wikidata, omitted P21 statements from entries for people whose sex or gender they could not determine. Unknown to Clements a bot created by Jura1 had been deployed that scanned biographical entries and used a known given name in an entry label to create a given name statement for that entry: for example, to give an item with the label ‘James M. Smith’ a statement using the property ‘given name’ (P735) and object ‘James’ (Q677191). The entries for given names contain ‘instance of’ statements that indicate the presumed gender of the given name: for example, ‘James’ (Q677191) is an ‘instance of’ (P31) a ‘male given name’ (Q12308941). The work of the first bot then triggered a second bot, created by Lockal, to add a P21 statement to any entry that included a given name statement whose object is given as an instance of ‘male given name’ (Q12308941) or ‘female given name’ (Q11879590). The overall effect was to assign statements about sex or gender to individuals for whom a sex or gender may have been assumed, but – based on research conducted by Clements – could not have been known, and should not have been assumed using a given name proxy. Potentially, these actions misgendered biographical entries on Wikidata.

Detached from the tacit knowledge that produced the P21 compromise in 2014, the use of P21 has – then – erred towards the classificatory logics of disciplinary systems that construct identity, towards the heteronormativity of the global north, towards binaries. It has also been a site of classificatory imperative that, as we saw with ‘ethnic group’ (P172), the Wikidata community has otherwise chosen to resist. Indeed, the permissible uses of another property relating to personal identity, ‘sexual orientation’ (P91), are similarly constrained, with evidence based on identity expression strongly preferred to external assignments of identity:

the sexual orientation of the person relative to their declared gender — use ONLY IF they have stated it themselves, unambiguously, or it has been widely agreed upon by historians after their death.

It might have been otherwise. *Homosaurus*, a controlled vocabulary of lesbian, qay, bisexual, transgender, queer/questioning, and others terms tackles the space inhabited by Wikidata P21 very differently. Created in 1997 by IHLIA LGBT Heritage as a Dutch and English gay and lesbian thesaurus,^6^ its latest update – v.3.2 – includes a range of narrower gender related terms from which to construct linked data biography. ‘Gender Identity’ (homoit0000571) allows for statements that capture individual experiences of gender as distinct from those statements that use the term ‘Gender Expression’ (homoit0000568) to capture perceptions or interpretations of an individual’s gender identity. And this term for expressed gender is distinct from ‘Assigned Gender’ (homoit0000078), which allows for statements that capture ascriptions of gender to individuals by parents or guardians or in legalised/medicalised settings (Homosaurus Vocabulary Site, 2021).

These narrower definitions draw attention to what we are doing with gender in *Beyond Notability*. We are dealing overwhelmingly in assignments of gender to adults, in gender as ascribed to historical agents in our sources and/or as perceived by us in our interpretation of those sources. We are not dealing with gender identity because for the majority of our subjects we have no direct access to their experiences of gender. And we are not dealing with gender expression, because that varies over time and between places, making our perceptions of gender a determinant of how we ascribe gender during our interpretation of our sources.

For example, in contemporaneous sources, the gender of nineteenth and early-twentieth century individuals is often signalled by the use of gendered honorifics. For many married women, conventions of polite society obscured how their personhood was recorded in the archival record, underscoring the historically specific heteronormative forces which controlled gender identity and made the gender binary resilient: for example, the given names of Mrs Edward Power (Q1389) and Mrs George Baldwinson (Q1381) are all lost, replaced by explicit assignments of gender through the institution of marriage. And in the cases of women recorded in the archival record with the gendered honorific ‘Miss’, a complex array of potential gender assignments are invoked, including pre-marital adolescence, unmarried womanhood, idiomatic sexual innuendo, and prudish allusions to homosexuality. In other sources the barriers and exclusions to which women were subject in nineteenth and early-twentieth century Britain provide indirect or implicit evidence for gender ascription. In the fields of archaeology, history, and heritage, women were excluded both from taking their degrees at certain universities and, until 1920, from being Fellows of the Society of Antiquaries. In the UK until 1918, the same group of people were excluded from suffrage if they were under 30 years old; a decade later all adults 21 years old or over were given equal voting rights. And once married, many women were limited in their ability to pursue certain kinds of work, either due to restrictions created by formal marriage bars or by conventions that intersected with class dynamics, industrial relations, and professional status (Glew, 2016). These examples are comparable to those used in 2013 by an unnamed Wikidata editor to reject Kaldari’s proposal to revert the label for P21 from ‘sex’ back to ‘gender’. But whereas for that editor who was and was not allowed be King of France had ‘nothing to do with’ gender, the narrower definitions of gender provided by *Homosaurus* and our readings of those definitions through the lens of *Beyond Notability*, suggests that P21 has everything to do with gender, and in particular ascriptions of gender, who gets to make those ascriptions, and the difficulties involved when restrictions based on those ascriptions lose credibility. In response, the equivalent property to P21 in the *Beyond Notability* knowledge base is used to record ‘assigned gender’, taking objects such as ‘woman’ and ‘man’ to indicate the perceptions of gender indicated by historical actors, assumed by administrative processes, or perceived by us as historians in our use of archival records (*Beyond Notability*, n.d.). Tracing the genealogy of the Wikidata property ‘sex or gender’ via these alternatives and lenses teases out how P21 works, how it has not only flattened sex and gender but also normalised the use of external ascriptions of gender based on essentialist perceptions of biology as determining gender and as unchanging over time and between places.

----

This article has considered Wikidata-as-infrastructure. It has explored our use of the *Beyond Notability* knowledge base as a lens through which to probe the conventions and assumptions that underpin the Wikidata data model, to articulate the community consensus and conventions that produce the model, and to understand how meaning has been made by classificatory logics spread across subject-predicate-object triples, property descriptions, and talk pages. We have argued that the Wikidata model favours a presentist and event-based temporality in ways that are ill-suited to the rich, subtle, and granular conceptions of historical and biographical change. We also argue that in its implementation, the Wikidata model tends to ascribe identity unambiguously and err towards heteronormative and disciplinary systems of constructing personhood. In so doing, we have drawn attention to areas where the Wikidata community’s imperatives towards classification and presentist viewpoints are unevenly applied, and in turn whose interests are served and oppressed by these consensus-based community conventions, by simplifications to biographical classification that make access and query services functional at scale. Wikidata is then as positionally and temporally ascribed as any infrastructure – the representations of identity that it produces cannot be taken unproblematically as a mirror of actions in the world. Therefore, whilst it is possible to input more data, to redress the underrepresentation of previously minoritised communities and demographics within this large-scale infrastructure, even after such inputs Wikidata-as-infrastructure is unlikely to adequately represent those communities: unless, that is, the community agrees to radical change.^7^

This work is significant because of the significant scale and reach of Wikidata. The infrastructure, inclusive of the community that build and maintain it, are a central node within our contemporary knowledge infrastructure ecosystem. Wikidata exemplifies the promise of the semantic web and in that spirit the Wikidata community has used linked data technologies to produce a model of humanity that people and machines can both learn from and contribute to. Wikidata is a remarkable achievement and yet one that has limitations that create and reinforce particular structures of power: it is inattentive to life course temporalities; it cannot accommodate unsettled and temporarily contingent relationships with nationhood, belonging, custom, place, and history; it is oriented around particular models of wage labour; and it breaks down when faced with the inadequacy of gender binaries. Of course, as a generalised linked data model, Wikidata cannot and should not be asked to model every intricacy of human life, past and present. However, in order to begin to fully understand their power in the world, especially when deployed at scale, we must seek to know the limitations of such models. As Geoffrey Bowker and Susan Leigh Star write ‘assigning things, people, or their actions to categories is a ubiquitous part of work in the modern, bureaucratic state’ (Bowker & Star, 2000: 285). Framing, testing, and challenging categorical work is in turn both a vital and enduring task.

## Data Availability

No datasets were generated or analysed during the current study.

## Footnotes

2 Our research for this article involved reading, analysing, and reproducing conversations and debates within the Wikidata community, much of which takes place on item ‘Talk’ pages. Wikidata advises contributors that ‘When you make a contribution to any Wikimedia Site, including on user or discussion pages, you are creating a permanent, public record of every piece of content added, removed, or altered by you’ (https://foundation.wikimedia.org/wiki/Policy:Privacy_policy#your-public-contribs (accessed 19 December 2023). These contributions are published under a Creative Commons Attribution-ShareAlike License. In our use of these contributions, we applied a reflexive ethics that seeks to minimise harm and to acknowledge our own standpoints. We were guided in so doing by aline shakti franzke, Anja Bechmann, Michael Zimmer, Charles M. Ess, and the Association of Internet Researchers (2020). *Internet Research: Ethical Guidelines 3.0*. https://aoir.org/reports/ethics3.pdf.

3 For Joshua Habgood-Coote, social practices around knowledge production are creating the conditions for an epistemic apocalypse (Habgood-Coote, 2023).

4 That is, items that contain an ‘instance of’ statement with the object ‘human’, so as to distinguish them from those biographical entries relating to fictional characters, non-human animals, and other biographical subjects.

5 Bowker and Star define torque as a term ‘used to describe the twisting that occurs when a formal classification system is mismatched with an individual’s biographical trajectory, memberships, or location’. Recovering the motivations behind classification of this kind typically involves analysing the use of infrastructures and projecting backward in time from occurrences of mismatch and torque. But in the case of Wikidata, the version control and talk pages embedded within its socio-technical infrastructure gives us direct – if always incomplete – evidence of how the naturalisation of political categories took place.

6 Based in Amsterdam (Netherlands), IHLIA stands for Internationaal Homo/Lesbisch Informatiecentrum en Archief.

7 Crystal Yragui has recently proposed that Wikidata stop making statements about sex and gender, though they acknowledge that the implementation of their suggestion was ‘not likely in the current environment’. As a compromise, they have proposed that the Wikidata community value accuracy over completeness (*Modelling Gender (Data Modelling Days 2023)*, 2023). This feels like a radical change and our research supports their proposal.

## References

[CR1] Agostinho, D. (2019). Archival encounters: Rethinking access and care in digital colonial archives. *Archival Science*, *19*(2), 141–165.

[CR2] Baker, J., Thornton, A., Harloe, K. (2024). Beyond Notability Knowledge Base. University of Southampton. https://eprints.soton.ac.uk/494228/ (accessed 8 October 2024).

[CR3] Bender, E. M., Gebru, T., McMillan-Major, A., et al. (2021). *On the dangers of Stochastic parrots* (p. 14). Can Language Models Be Too Big?.

[CR4] Beyond Notability (n.d.). Assigned gender (p3). Retrieved October 8, 2024a, from https://beyond-notability.wikibase.cloud/wiki/Property:P3

[CR5] Beyond Notability (n.d.). Dorothy Nancy Stroud (Q1695). https://beyond-notability.wikibase.cloud/wiki/Item:Q1695 (accessed 19 December 2023b).

[CR6] Beyond Notability (n.d.). Edith Bradley (Q668). Retrieved December 19, 2023c, from https://beyond-notability.wikibase.cloud/wiki/Item:Q668

[CR7] Borgman, C. L. (2015). *Big Data, Little Data, No Data: Scholarship in the Networked World*. Cambridge, Massachusetts: The MIT Press. https://cornell-library.skillport.com/skillportfe/main.action?assetid=82612 (accessed 12 January 2024).

[CR8] Bowker, G. C., & Star, S. L. (2000). *Sorting things out: Classification and its consequences*. The MIT Press.

[CR9] Braudel, F. (1972). *The Mediterranean: And the Mediterranean World in the Age of Philip II. Volume l*. Second revised edition. London: Collins.

[CR10] Bressey, C. (2006). Invisible Presence: The Whitening of the Black Community in the Historical Imagination of British Archives. *Archivaria*: 47–61.

[CR11] Candela, G., Pereda, J., Sáez, D. (2023). An ontological approach for unlocking the Colonial Archive. *Journal on Computing and Cultural Heritage*. Epub ahead of print 28 April 2023. 10.1145/3594727

[CR12] Causevic, A., Philip, K., Zwick-Maitreyi, M. (2020). Centering knowledge from the margins: our embodied practices of epistemic resistance and revolution. *International Feminist Journal of Politics, 22*(1). Routledge: 6–25.

[CR13] Clements, C. (2021). User talk:Jura1/D/183/D/1aruJ:klat resU - Gendered first names added by bot. *Wikidata*. https://www.wikidata.org/wiki/User_talk:Jura1/D/183/D/1aruJ:klat_resU (accessed 2 January 2024).

[CR15] D’Ignazio, C., & Klein, L. F. (2020). *Data Feminism*. Cambridge, Massachusetts: The MIT Press. https://ebookcentral.proquest.com/lib/suss/detail.action?docID=6120950

[CR14] Dhaliwal, R. S. (2022). On Addressability, or What Even Is Computation? *Critical Inquiry, 49*(1). The University of Chicago Press: 1–27.

[CR16] Emw (2014). Wikidata:Project chat/Archive/2014/07 - Gender redundancy. *Wikidata*. https://www.wikidata.org/wiki/Wikidata:Project_chat/Archive/2014/07#Gender_redundancy (accessed 2 January 2024).

[CR17] Evenstein Sigalov, S., & Nachmias, R. (2023). Investigating the potential of the semantic web for education: Exploring Wikidata as a learning platform. *Education and Information Technologies, 28*. 10.1007/s10639-023-11664-1 PMC10009355 37361737

[CR18] Filceolaire (2014). Wikidata:Project chat/Archive/2014/07 - Gender redundancy. *Wikidata*. https://www.wikidata.org/wiki/Wikidata:Project_chat/Archive/2014/07#Gender_redundancy (accessed 2 January 2024).

[CR19] Gebru, T., Bender, E. M., McMillan-Major, A. (2023). Statement from the listed authors of Stochastic Parrots on the AI pause letter. In: *The DAIR Institute*. https://www.dair-institute.org/blog/letter-statement-March2023 (accessed 1 April 2023).

[CR20] Gilroy, P. (2000). *Against race: Imagining Political Culture beyond the Color line*. Belknap Press of Harvard University.

[CR21] Glew, H. (2016). *Gender, rhetoric and regulation: Women’s work in the Civil Service and the London County Council, 1900-55*. Manchester University.

[CR22] Graham, M., Straumann, R. K., & Hogan, B. (2015). Digital Divisions of Labor and Informational Magnetism: Mapping Participation in Wikipedia. *Annals of the Association of American Geographers* 105(6). Routledge: 1158–1178.

[CR23] Groebner, V. (2007). *Who are you? Identification, deception, and Surveillance in Early Modern Europe*. MIT Press.

[CR24] Habgood-Coote, J. (2023). Deepfakes and the epistemic apocalypse. *Synthese*, *201*(3), 103.

[CR25] Hall, S. (2017). *Familiar Stranger: A Life between Two Islands* (ed. B Schwarz). Stuart Hall: selected writings. Durham: Duke University Press. http://site.ebrary.com/id/11365661 (accessed 30 January 2024).

[CR26] Hill, K. (2016). *Women and museums 1850–1914: Modernity and the gendering of knowledge*. Manchester University.

[CR27] Homosaurus Vocabulary Site. (2021). *Homosaurus Vocabulary Site* (2021). https://homosaurus.org/v3 (accessed 22 January 2024).

[CR28] Jacques, M. (2018). Hall, Stuart McPhail (1932–2014), cultural theorist and political commentator. *Oxford Dictionary of National Biography*. https://www.oxforddnb.com/view/10.1093/odnb/9780198614128.001.0001/odnb-9780198614128-e-107673 (accessed 4 August 2022).

[CR29] Jheald (2018). Property talk:P27 - Multiple UK values - reality check pls. *Wikidata*. https://www.wikidata.org/wiki/Property_talk:P27#Multiple_UK_values_-_reality_check_pls (accessed 11 October 2022).

[CR30] Kaldari (2013). Property talk:P21/Archive 1 - Rename (en) label ‘sex’->’gender’. *Wikidata*. https://www.wikidata.org/wiki/Property_talk:P21/Archive_1#Rename_(en)_label_’sex’-%3E’gender’ (accessed 2 January 2024).

[CR31] Langhamer, C. (2017). Feelings, women and work in the long 1950s. *Women’s History Review*, *26*(1), 77–92.

[CR32] Lavorgna, A., & Ugwudike, P. (2021). The datafication revolution in criminal justice: An empirical exploration of frames portraying data-driven technologies for crime prevention and control. *Big Data* & *Society, 8*(2). SAGE Publications Ltd: 20539517211049670.

[CR33] McCarthy, H. (2021). *Double lives: A history of Working Motherhood in Modern Britain*. Bloomsbury Publishing.

[CR34] Mirzoeff, N. (2023). *White Sight: Visual politics and practices of Whiteness*. The MIT Press.

[CR35] Modelling Gender *(Data Modelling Days 2023) (2023)*. https://www.youtube.com/watch?v=AkBD5AKmY0M (accessed 19 December 2023).

[CR36] Noble, S. U. (2018). *Algorithms of Oppression: How Search Engines Reinforce Racism*. New York: New York University Press. https://ebookcentral.proquest.com/lib/suss/detail.action?docID=4834260 10.1126/science.abm5861 34709921

[CR37] Pellissier Tanon, T., & Suchanek, F. (2019). Querying the Edit History of Wikidata. In: Hitzler P, Kirrane S, Hartig O, et al. (Eds.) *The Semantic Web: ESWC 2019 Satellite Events*. Lecture Notes in Computer Science. Cham: Springer International Publishing, pp. 161–166. http://link.springer.com/10.1007/978-3-030-32327-1_32 (accessed 19 December 2023).

[CR38] PinkAmpersand (2013). Property talk:P27 - Renaming. *Wikidata*. https://www.wikidata.org/wiki/Property_talk:P27#Renaming (accessed 11 October 2022).

[CR39] Piscopo, A. (2019). *Structuring the world’s knowledge: socio-technical processes and data quality in Wikidata*. PhD. University of Southampton. https://eprints.soton.ac.uk/438873/ (accessed 4 August 2022).

[CR40] Piscopo, A., & Simperl, E. (2018). Who Models the World? Collaborative Ontology Creation and User Roles in Wikidata. *Proceedings of the ACM on Human-Computer Interaction* 2(CSCW): 1–18.

[CR41] Rutz, A., Sorokina, M., Galgonek, J. (2022). The LOTUS initiative for open knowledge management in natural products research. *eLife* Donoso DA, Akhmanova A, and Tapley Hoyt C (Eds.) 11. eLife Sciences Publications, Ltd: e70780. 10.7554/eLife.70780 PMC9135406 35616633

[CR42] Sengupta, A. (2021). Decolonising Wikidata: why does knowledge justice matter for structured data? In: *WikidataCon 2021*, 2021. https://www.youtube.com/watch?v=wn2BrQomvFU (accessed 4 August 2022).

[CR43] Sengupta, A., & Bouterse, S. (2017). Research – Whose Knowledge? http://whoseknowledge.org/why/ (accessed 7 September 2017).

[CR44] Seth, S. (2009). Putting knowledge in its place: Science, colonialism, and the postcolonial. *Postcolonial Studies*, *12*(4), 373–388.

[CR45] Sex (P21) (2013). https://www.wikidata.org/w/index.php?title=Property:P21%26oldid=5827730

[CR46] Star, S. L. (1990). Power, Technology and the Phenomenology of Conventions: On being Allergic to Onions. *The Sociological Review* 38(1). SAGE Publications Ltd: 26–56.

[CR47] Stevenson, A. (2019). *Scattered finds: Archaeology, Egyptology and museums*. UCL.

[CR48] Thornton, A. (2018). *Archaeologists in print: Publishing for the people*. UCL.

[CR49] Thylstrup, N. B., Agostinho, D., Ring, A., et al. (2021). *Uncertain archives critical keywords for Big Data*. The MIT Press.

[CR50] Tobias1984 (2013). Property talk:P21/Archive 1 - Rename (en) label ‘sex’->’gender’. *Wikidata*. https://www.wikidata.org/wiki/Property_talk:P21/Archive_1#Rename_(en)_label_’sex’-%3E’gender’ (accessed 2 January 2024).

[CR51] Tripodi, F. (2021). Ms. Categorized: Gender, notability, and inequality on Wikipedia. *New Media* & *Society*. SAGE Publications: 14614448211023772.

[CR52] Uses of P172 (n.d.). Available at: https://w.wiki/5ZUMaccessed 3 November (2023).

[CR53] Vrandečić, D., Pintscher, L., & Krötzsch, M. (2023). Wikidata: The Making Of. In: *Companion Proceedings of the ACM Web Conference 2023*, Austin TX USA, 30 April 2023, pp. 615–624. ACM. 10.1145/3543873.3585579 (accessed 25 May 2023).

[CR54] Wikidata (2013a). country of citizenship (P27). https://www.wikidata.org/wiki/Property:P27 (accessed 11 October 2022).

[CR55] Wikidata (2013b). gender (P21). https://www.wikidata.org/w/index.php?title=Property:P21%26oldid=5797363

[CR56] Wikidata (2013c). gender (P21). https://www.wikidata.org/w/index.php?title=Property:P21%26oldid=5800916

[CR57] Wikidata (2013d). Property talk:P21/Archive 1 - Rename (en) label ‘sex’->’gender’. https://www.wikidata.org/wiki/Property_talk:P21/Archive_1#Rename_(en)_label_’sex’-%3E’gender’ (accessed 2 January 2024).

[CR58] Wikidata (2013e). sex (or gender) (P21). https://www.wikidata.org/w/index.php?title=Property:P21%26direction=next%26oldid=96205268 (accessed 20 December 2023).

[CR59] Wikidata (2013f). sex (P21). https://www.wikidata.org/w/index.php?title=Property:P21%26direction=next%26oldid=96205078 (accessed 20 December 2023).

[CR60] Wikidata (2013g). Property talk:P21/Archive 1 - Rename (en) label ‘sex’->’gender’. https://www.wikidata.org/wiki/Property_talk:P21/Archive_1#Rename_(en)_label_’sex’-%3E’gender’ (accessed 2 January 2024).

[CR61] Wikidata (2013h). Property talk:P21/Archive 1 - What do you enter when sex and gender do not agree? https://www.wikidata.org/wiki/Property_talk:P21/Archive_1#What_do_you_enter_when_sex_and_gender_do_not_agree? (accessed 2 January 2024).

[CR62] Wikidata (2014). Sex or gender (P21). https://www.wikidata.org/wiki/Property:P21 (accessed 2 January 2024).

[CR63] Wikidata (2015). Property talk:P27 - Use of this property in the scope of history. https://www.wikidata.org/wiki/Property_talk:P27#Use_of_this_property_in_the_scope_of_history (accessed 11 October 2022).

[CR64] Wikidata (2019). Wikidata talk:WikiProject LGBT/Archive/2019 - Sex or gender data model. https://www.wikidata.org/wiki/Wikidata_talk:WikiProject_LGBT/Archive/2019#Sex_or_gender_data_model (accessed 2 January 2024).

[CR65] Wikidata (2023). Property talk:P108 - employer. https://www.wikidata.org/wiki/Property_talk:P108 (accessed 19 December 2023).

[CR66] Wikidata (n.d.) Angela Merkel (Q567). https://www.wikidata.org/wiki/Q567 (accessed 19 December 2023a).

[CR67] Wikidata (n.d.). Database reports/Complex constraint violations/P172. Retrieved October 11, 2022b, from https://www.wikidata.org/wiki/Wikidata:Database_reports/Complex_constraint_violations/P172#Claims_without_source

[CR68] Wikidata (n.d.). Neil Armstrong (Q1615). Retrieved December 19, 2023c, from https://www.wikidata.org/wiki/Q1615

[CR69] Wikidata (n.d.). Olwen Brogan (Q26727497). Retrieved December 19, 2023d, from https://www.wikidata.org/wiki/Q26727497

[CR70] Wikidata (n.d.). Stuart Hall. Retrieved October 11, 2022e, from https://www.wikidata.org/wiki/Q450741

[CR71] Wikidata Query Service (n.d.). Values used by property P21. December 20, 2023, from https://w.wiki/Qua

[CR72] Wikipedia (2004). WikiProject Ethnic groups. https://en.wikipedia.org/w/index.php?title=Wikipedia:WikiProject_Ethnic_groups%26oldid=2097311 (accessed 11 October 2022).

[CR73] Willaert, T., & Roumans, G. (2020). Nitpicking online knowledge representations of governmental leadership. The case of Belgian prime ministers in Wikipedia and Wikidata. *LIBER Quarterly*, *30*(1), 1.

[CR74] Zolo (2013). Property talk:P27 - old times. *Wikidata*. https://www.wikidata.org/wiki/Property_talk:P27#old_times (accessed 11 October 2022).

